# Pectus Excavatum in an Adolescent Swimmer: Integrating Thoracic Morphology and Cardiopulmonary Exercise Testing

**DOI:** 10.3390/jfmk11040415

**Published:** 2026-10-10

**Authors:** Vasileios T. Stavrou, Charalampos Charalampidis

**Affiliations:** Medical School, University of Cyprus, 1678 Nicosia, Cyprus; charalampidis.charalampos@ucy.ac.cy

**Keywords:** functional morphology, echocardiography, cardiopulmonary exercise testing

## Abstract

**Background and Objectives**: Pectus excavatum (PE) alters thoracic geometry and may affect cardiac filling and respiratory mechanics. However, anatomical severity does not necessarily lead to functional limitations. This case study examined the integrated physiological response from rest to maximal exercise and recovery in an asymptomatic, competitive adolescent swimmer with PE. **Methods**: A 16-year-old male competitive swimmer with asymmetric pectus excavatum (Park type 2A; Haller index 3.3) underwent pulmonary function testing and resting two-dimensional echocardiography, as well as cardiopulmonary exercise testing. **Results**: From rest to maximal exercise, VO_2_ increased approximately 5.9-fold, from 576 to 3378 mL·min^−1^ (45.0 mL·kg^−1^·min^−1^; 92% predicted; AT: 41.2 mL·kg^−1^·min^−1^), while oxygen pulse increased from 6.0 to 17.9 mL O_2_·beat^−1^ (AT: 17.0 mL O_2_·beat^−1^) and OB from 30.3 to 91.3 mL O_2_·breath^−1^ (AT: 83.5 mL O_2_·breath^−1^). PQR was 5.05 at rest and 5.11 beats·breath^−1^ at peak (AT: 4.92 beats·breath^−1^), while peak V_E_/MVV reached 56.0% (AT: 48.6%). Pulmonary function testing showed a forced vital capacity at 120% of predicted, forced expiratory volume in one second at 119% of the predicted value and maximal inspiratory pressure was 120% of the predicted value. Resting echocardiography revealed preserved left ventricular longitudinal myocardial deformation (GLS −21.8%) and a transmitral E/A ratio of 1.46. **Conclusions**: Despite having a thoracic deformity, the athlete demonstrated preserved resting function and an integrated cardiopulmonary response to maximal exercise, with no ventilatory limitations.

## 1. Introduction

Pectus excavatum (PE) is characterised by the posterior displacement of the sternum and adjacent costal cartilages. This reduces the anteroposterior dimension of the thorax and alters the spatial relationship between the chest wall, heart, and lungs. As the right ventricle is located directly behind the sternum, a reduction in retrosternal space may affect right-sided cardiac filling. Additionally, regional chest wall restriction can modify thoracoabdominal motion and ventilatory mechanics [1,2]. The Haller index (HI) quantifies thoracic geometry, but does not directly measure cardiac output, ventilatory reserve, or exercise capacity. Previous resting and exercise studies have demonstrated heterogeneous functional findings in individuals with PE [3,4]. This distinction is particularly relevant in asymptomatic athletes, for whom preserved resting measurements do not necessarily reflect physiological reserve during strenuous exercise.

Resting echocardiography provides information on baseline cardiac structure, myocardial deformation, and diastolic filling, whereas cardiopulmonary exercise testing (CPET) extends this assessment by characterising the integrated physiological response to increasing metabolic demand. CPET-derived variables such as oxygen uptake (VO_2_), oxygen pulse, ventilatory response and recovery kinetics provide additional information on cardiovascular oxygen delivery, peripheral oxygen utilisation and ventilatory recruitment [5,6,7]. However, oxygen pulse should be interpreted as an integrated variable influenced by both stroke-volume response and peripheral arteriovenous oxygen extraction, rather than as a direct measure of stroke volume. Exercise and recovery are also accompanied by autonomic adjustments. However, autonomic function itself cannot be directly quantified using routine CPET or resting echocardiography [8,9]. The added value of this case lies in its integrated evaluation of thoracic morphology, resting pulmonary and myocardial function, and the continuous physiological trajectory from rest to early recovery, including the anaerobic threshold and peak exercise, in an asymptomatic, competitive adolescent swimmer.

This case report aimed to interpret the functional significance of a defined thoracic deformity in relation to exercise physiology. More specifically, the physiological response from rest to peak exercise and the initial recovery period was characterised using variables derived from CPET, with pulmonary function testing and resting echocardiography providing the baseline cardiopulmonary phenotype for interpretation.

## 2. Materials and Methods

### 2.1. Case Design

This descriptive case report concerns a 16-year-old male competitive swimmer with asymmetric pectus excavatum who was evaluated as part of the pre-participation screening process. This included an assessment of the anatomy of the thorax, testing of pulmonary function at rest, transthoracic echocardiography and CPET. The athlete had eight years of competitive swimming experience and trained five times per week, totalling around 10 h·week^−1^, primarily in the afternoon. He regularly participated in competitive swimming events and primarily competed in short-distance freestyle events (50–100 m). The athlete was not receiving any regular medication at the time of assessment. The athlete was instructed to refrain from strenuous exercise for at least 24 h prior to the assessment. His thoracic morphology was classified as Park type 2A, based on the symmetry and distribution of the anterior chest-wall depression [10]. Previous clinical documentation of the athlete revealed a Haller Index (HI) of 3.3, deter-mined using an imaging-based assessment. The HI is defined as the ratio of the maximum internal transverse thoracic diameter to the minimum anteroposterior distance between the posterior surface of the sternum and the anterior surface of the vertebral body [11]. These classifications were used solely to characterise thoracic morphology and anatomical severity and were not interpreted as direct measures of cardiopulmonary function.

### 2.2. Pulmonary Function Testing

Spirometry was performed before CPET using a MasterScreen-CPX pneumotachograph system (VIASYS Healthcare, Höchberg, Germany), in accordance with ATS/ERS recommendations [12]. Three maximal flow–volume manoeuvres were performed in the seated position, with approximately 40 s of rest between each one, and the manoeuvre that was technically the most acceptable was selected for analysis. The following were recorded: forced vital capacity (FVC), forced expiratory volume in 1 s (FEV_1_), and the FEV_1_/FVC ratio. Estimated maximum voluntary ventilation (MVV) was calculated from the best absolute FEV_1_ value using the equation MVV = FEV_1_ × 35 and was expressed in L·min^−1^. Predicted spirometric values were calculated using the reference equations of Zapletal et al. for children and adolescents [13]. Respiratory muscle strength was also assessed using maximal inspiratory pressure (PImax), which was measured as maximal inspiratory mouth pressure using a MicroRPM portable respiratory pressure meter (CareFusion, San Diego, CA, USA) in the seated position and expressed in cm H_2_O according to the previously described methodology [14]. The predicted PImax was calculated according to the method of Black and Hyatt [15], using the following equation for males: 143 − (0.55 × age).

### 2.3. Echocardiographic Assessment

Two-dimensional resting transthoracic echocardiography was performed on the athlete in the left lateral decubitus position using a Vivid BT08 ultrasound system (General Electric, Miami, FL, USA). Standard parasternal long-axis and short-axis views, as well as apical four- and two-chamber views, were obtained in accordance with established paediatric and chamber-quantification recommendations [16,17]. Left ventricular longitudinal myocardial deformation was assessed using two-dimensional speckle-tracking echocardiography. Global longitudinal strain (GLS) was calculated from the apical long-axis, four-chamber and two-chamber views available and expressed as a negative percentage. Early (E) and late (A) transmitral diastolic velocities were obtained using pulsed-wave Doppler and the E/A ratio was calculated. GLS was interpreted as a measure of left ventricular longitudinal myocardial deformation, rather than as a surrogate for exercise stroke volume or right ventricular function [18].

### 2.4. Cardiopulmonary Exercise Testing

Cardiopulmonary exercise testing was performed on an electronically braked Ergoselect 100 cycle ergometer (ergoline GmbH, Bitz, Germany) connected to a MasterScreen CPX metabolic system (VIASYS HealthCare, Höchberg, Germany), using a protocol previously applied by our group [6,19]. Respiratory gas exchange was measured breath-by-breath, including oxygen uptake (VO_2_, mL·min^−1^), carbon dioxide output (VCO_2_, mL·min^−1^), minute ventilation (V_E_, L·min^−1^), and breathing frequency (f_B_ breaths·min^−1^). Heart rate (HR, beats·min^−1^) was continuously monitored by 12-lead electrocardiography (CareFusion, San Diego, CA, USA), and peripheral oxygen saturation (SpO_2_) by pulse oximetry. The CPET protocol comprised four consecutive stages: Stage 1. Rest: 2 min at 0 W and 0 rpm. Stage 2. Warm-up and familiarisation: 3 min of unloaded cycling at 55–60 rpm. Stage 3. Incremental exercise: exercise commenced at 20 W, with work rate increasing continuously by 20 W·min^−1^ in a ramp pattern while cadence was maintained at 60–65 rpm and continued until volitional exhaustion. Stage 4. Recovery: 5 min of unloaded cycling at a self-selected cadence below 50 rpm. Age-predicted maximal heart rate was calculated for descriptive purposes using the Tanaka equation: 208 − (0.7 × age) [20].

For the present case analysis, the anaerobic threshold was manually re-evaluated by inspecting the original gas-exchange response in more detail. This assessment considered the disproportionate increase in VCO_2_ compared to VO_2_, as well as the accompanying ventilatory and end-tidal gas responses.

Lower-limb fatigue upon termination of exercise was evaluated using the Borg CR10 scale. Peak exercise was defined as the highest workload and oxygen uptake achieved during exercise that was limited by symptoms. Predicted peak VO_2_ and peak oxygen pulse values were derived from the paediatric reference equations of Cooper et al. [21,22]. For boys, predicted peak VO_2_ was calculated as 52.8 × body mass (kg) − 303.4, and predicted peak oxygen pulse as 0.23 × height (cm) − 24.4.

To characterise the integrated physiological response from rest to maximal exercise, additional CPET-derived variables were calculated from temporally aligned measurements according to our previously described approach [6,7]:

Oxygen breath (OB) $=\frac{VO_{2}}{f_{B}}$ and was expressed as mL O_2_·breath^−1^.

Pulse–respiration quotient (PQR) $=\frac{\mathrm{HR}}{f_{B}}$ and was expressed as beats·breath^−1^.

Oxygen pulse $=\frac{VO_{2}}{\mathrm{HR}}$ and was expressed as mL O_2_·beat^−1^.

Ventilatory utilisation was expressed as V_E_/MVV (%), using the estimated MVV derived from resting FEV_1_. These variables were examined across the exercise trajectory from rest to the anaerobic threshold, maximal exercise, and recovery to describe the integrated interaction between oxygen uptake, cardiovascular response, breathing pattern, and ventilatory reserve.

## 3. Results

**Figure 1:**
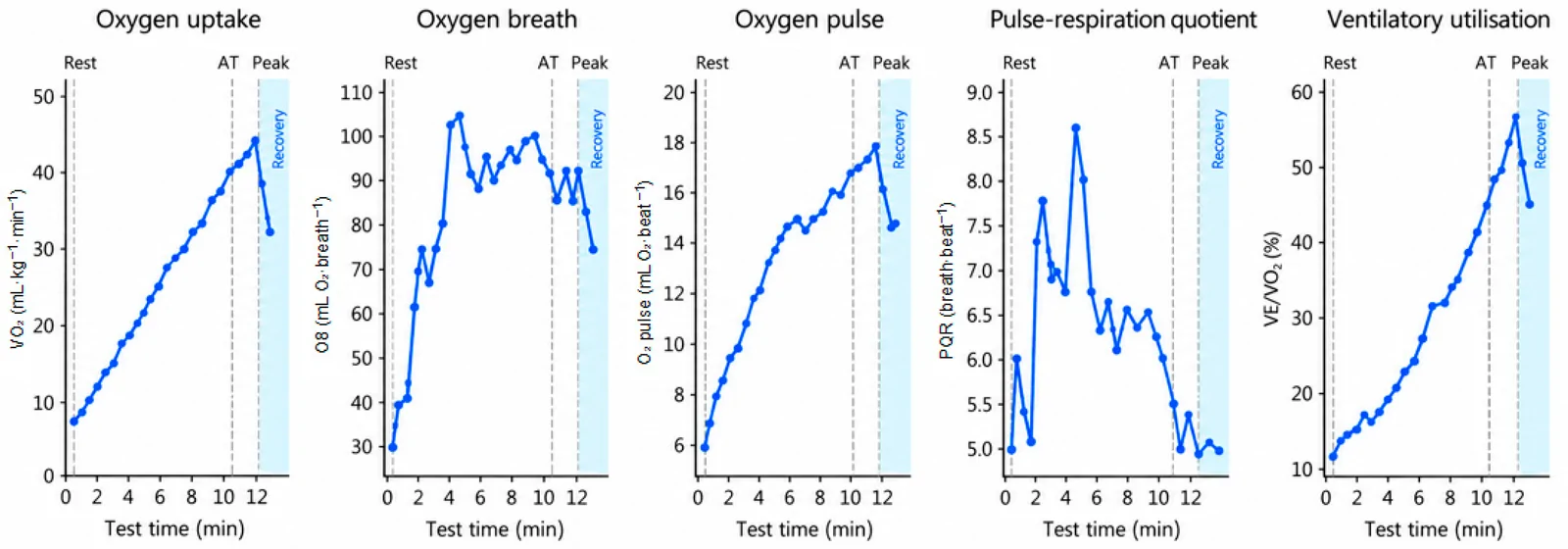
Continuous CPET responses from rest (0 W), anaerobic threshold (AT, 236 W) to peak exercise (260 W) and first minute of recovery (0 W).

The athlete presented with eccentric, asymmetric pectus excavatum (Park type 2A; Haller index 3.3), was asymptomatic during daily activities and training, and had a body mass index of 23.9 kg·m^−2^ and a body surface area of 1.92 m^2^ at CPET. Resting pulmonary function tests showed that pulmonary function was preserved, with FVC of 5.71 L (120% of predicted), FEV_1_ of 4.64 L (119% of predicted), FEV_1_/FVC of 81.35%, MVV of 162.4 L·min^−1^ and PImax of 161 cmH_2_O (120% of predicted). Resting echocardiography showed a left ventricular global longitudinal strain (GLS) of −21.8%, a transmitral E/A ratio of 1.46, and no mitral valve prolapse was reported. During incremental exercise, VO_2_ increased from 576 mL·min^−1^ (7.7 mL·kg^−1^·min^−1^) at rest to 3091 mL·min^−1^ at the anaerobic threshold (41.2 mL·kg^−1^·min^−1^; 84.5% of predicted peak VO_2_) and 3378 mL·min^−1^ at peak exercise (45.0 mL·kg^−1^·min^−1^; 92% predicted). Along the same trajectory, the oxygen pulse increased from 6.0 to 17.0 and 17.9 mL O_2_·beat^−1^ (110% of the predicted maximum value), the OB increased from 30.3 to 83.5 and 91.3 mL O_2_·breath^−1^. The progressive increase in VE/MVV reflected the expected rise in ventilatory demand with exercise, while the peak value of 56.0% corresponded to a calculated breathing reserve of 44.0%. Meanwhile, the PQR remained similar at the three physiological landmarks (5.05, 4.92 and 5.11 beats·breath^−1^, respectively). The exercise session was terminated due to lower-limb fatigue (Borg CR10 = 7). The peak heart rate was 189 beats per minute, the peak VE was 91 litres per minute, the peak fB was 37 breaths per minute, the peak RER was 1.04, and the peak SpO_2_ was 99%, with an estimated breathing reserve of 44.0%. During the initial unloaded recovery period, the main physiological parameters decreased simultaneously: at 20 s post-peak, VO_2_ was 3079 mL·min^−1^, the oxygen pulse was 16.7 mL O_2_·beat^−1^, the OB was 85.5 mL O_2_·breath^−1^, the VE/MVV was 48.6%, and the heart rate was 184 beats/min. Further reductions occurred by 50 s, including a decrease in the VE/MVV to 46.2% and the PQR to 5.00 beats·breath^−1^. Figure 1 shows the continuous response from rest through to maximal exercise and first minute of recovery.

## 4. Discussion

**Figure 2:**
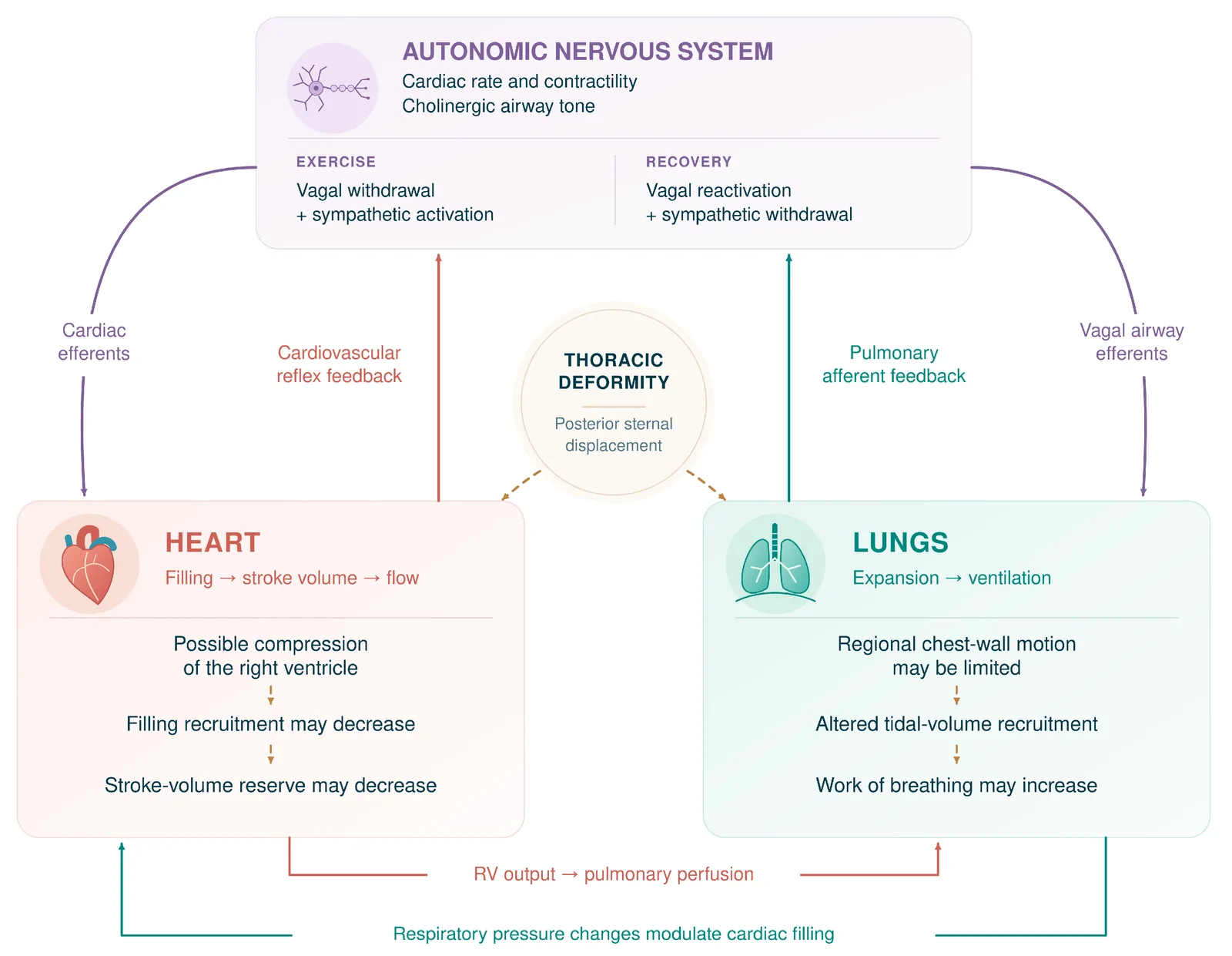
This is a mechanistic framework that links thoracic morphology to cardiac, respiratory, and autonomic physiology. Solid arrows indicate established physiological relationships, while dashed ochre arrows indicate the potential effects of pectus excavatum. Purple arrows represent autonomic neural pathways, red arrows indicate cardiovascular interactions, and teal arrows represent respiratory interactions. RV = right ventricle; PE = pectus excavatum.

This case study demonstrates how a structural chest wall deformity can be understood in terms of dynamic physiology. Resting pulmonary function and echocardiographic myocardial mechanics establish the baseline phenotype, while CPET shows how the body responds from rest to maximal exercise and recovery. The athlete had an eccentric, asymmetric pectus excavatum (Park type 2A; HI 3.3). Posterior sternal displacement may reduce the retrosternal space, thereby influencing right ventricular filling, while chest wall asymmetry may alter regional respiratory mechanics [1,2]. However, anatomical severity alone does not determine functional impairment, as suggested by Choi et al., who found no consistent association between HI and resting measures of right ventricular function [3]. In this athlete, pulmonary volumes and inspiratory muscle strength were approximately 20% above predicted values, while resting GLS and transmitral filling were normal, indicating no overt pulmonary, inspiratory muscle or LV myocardial impairment at rest. From rest to peak exercise, VO_2_ increased by 2802 mL·min^−1^, oxygen pulse by 11.9 mL O_2_·beat^−1^, and OB by 61.0 mL O_2_·breath^−1^, whereas PQR changed minimally (+0.06 beats·breath^−1^). This pattern indicates coordinated recruitment of oxygen transport, cardiovascular response, and breathing as metabolic demand increased. The progressive increase in oxygen pulse to 17.9 mL O_2_·beat^−1^ (110% predicted) was consistent with a preserved cardiovascular response. The oxygen pulse reflects the combined influence of stroke volume and peripheral oxygen extraction and can be considered an indirect measure of the stroke volume response. Ventilation increased during maximal exercise, with peak VE reaching 56.0% of MVV. Alongside preserved resting spirometry and maintained oxygen saturation, these results suggest that ventilatory capacity is intact, with no evidence of limitation. Heart rate increased by 93 beats·min^−1^ from rest to peak and then decreased by 24 beats per minute within the first minute of recovery. This pattern was consistent with the expected autonomic adjustments during exercise and recovery, although autonomic function was not assessed directly [8,9]. Figure 2 summarises the potential interactions between thoracic morphology and cardiorespiratory regulation but does not imply that autonomic mechanisms were directly measured in this athlete. This physiological profile is similar to the preserved-function phenotype described by Satur et al. [4] but differs from the lower VO_2_ peak and oxygen pulse reported by Casatori et al. [23], as well as the exercise-related RV abnormalities described by Raggio et al. [1]. Taken together, these findings emphasise the physiological diversity of PE and advocate for a personalised approach that considers thoracic morphology alongside resting cardiopulmonary function, dynamic exercise responses and neural regulation, rather than focusing solely on anatomical severity.

### Limitations

Several limitations should be acknowledged. Supine FVC was not assessed. Exercise echocardiography was unavailable for evaluating dynamic cardiac responses and thoracic CT imaging was unavailable for assessing potential additional intrathoracic anatomical abnormalities.

## 5. Conclusions

This case demonstrates that the functional significance of pectus excavatum cannot be determined by examining thoracic morphology alone. Combining resting cardiac and pulmonary assessments with CPET provides a more meaningful evaluation of how deformity affects physiology as exercise intensity increases.

## Data Availability

All data are available after request.
